# Splicing dependency between *EIF4G2* introns is mediated by exon definition and relies on a downstream splicing event

**DOI:** 10.1016/j.jbc.2026.113304

**Published:** 2026-06-27

**Authors:** Marie-Lee Pelletier, Joëlle Vincent, Téa Malo, Camille Séguin, Volodymyr Tsybulskyi, Egor Semenchenko, Ibrahim Soumana Adamou, Irmtraud M. Meyer, Karine Choquet

**Affiliations:** 1 Department of Biochemistry and Functional Genomics, Faculty of Medicine and Health Sciences, Université de Sherbrooke, Sherbrooke, Canada; 2 Research Center on Aging, CIUSSS de l’Estrie-CHUS, Sherbrooke, Canada; 3 Berlin Institute for Medical Systems Biology, Max Delbrück Centre, Berlin, Germany; 4 Freie Universität Berlin, Department of Biology, Chemistry and Pharmacy, Institute of Chemistry and Biochemistry, Berlin, Germany; 5 Freie Universität Berlin, Department of Mathematics and Computer Science, Institute of Computer Science, Berlin, Germany

**Keywords:** exon definition, minigene, RNA splicing, splicing coordination, splicing order

## Abstract

In mammalian cells, pre-mRNA splicing requires the excision of multiple introns within the same nascent transcript through the coordinated action of the spliceosome and accessory factors. We and others recently demonstrated that multi-intron splicing follows a predetermined order, and that removal of proximal introns is coordinated. Notably, specific introns that are removed later within a transcript depend on their neighboring introns for efficient excision, but the underlying mechanisms remain unknown. Here, we investigated the determinants of splicing order and coordination using a multi-intron minigene splicing reporter based on the human gene *EIF4G2*. We found that dependency between a later-excised intron and its neighbor is partially mediated through the downstream 5′ splice site, consistent with the exon definition model, but is independent from intron length. Importantly, splicing coordination also relies on a prior downstream act of splicing, but not on specific *cis* regulatory elements in neighboring introns. Together, these findings improve our understanding of how distinct splicing reactions are connected and coordinated to produce mature RNA molecules.

Pre-mRNA splicing is an essential step of mammalian gene expression that is catalyzed by the spliceosome, a large complex composed of five distinct small nuclear ribonucleoproteins (snRNPs). Spliceosome assembly is a stepwise process that begins with binding of the U1 snRNP to the 5′ splice site, followed by association of U2AF1 and U2AF2 to the 3′ splice site and polypyrimidine tract. Next, U2 snRNP binds to the branch point sequence near the 3′ splice site. The tri-snRNP, composed of U4, U5 and U6 snRNPs, is then recruited to form the pre-B complex, which transitions to the B complex after removal of U1 snRNP, followed by additional rearrangements that lead to catalytic activation (1). Direct interactions between RNA polymerase (Pol) II and U1 snRNP or U2AF1 suggest that spliceosome assembly initiates co-transcriptionally (2, 3), while intron excision can occur co- or post-transcriptionally in mammalian cells (reviewed in (4, 5)).

Human genes contain many long introns, yet splicing mechanisms have been mostly studied using synthetic pre-mRNAs (6, 7, 8, 9) or minigene constructs (10, 11, 12, 13, 14, 15, 16) consisting of one or two introns. However, increasing evidence indicates that splicing reactions within a pre-mRNA do not occur independently from one another. Mutation of a 5′ splice site impairs excision of the upstream intron and/or causes skipping of the adjacent exon (17, 18) and *in vitro* binding of U2 snRNP to a 3′ splice site is enhanced if U1 snRNPs are bound to the upstream and downstream 5′ splice sites (6, 19). Splicing rates and efficiencies tend to be coordinated across introns from the same gene (20, 21), while long-read sequencing studies suggest that excision of proximal introns within single pre-mRNAs is synergistic (22, 23). Furthermore, multiple studies have shown that splicing of mammalian pre-mRNAs does not proceed sequentially from the 5′ to the 3′ end (10, 18, 22, 24, 25, 26, 27, 28). Instead, splicing occurs in a predetermined order that is the same across transcripts from the same gene and is primarily determined by *cis* regulatory features (10, 18, 22, 27, 28, 29). Notably, local “slowpoke” introns were defined as introns that are always excised after their upstream and downstream neighbors (10). In minigene splicing reporter assays, local slowpoke introns had low excision efficiencies when tested alone, while addition of their upstream and/or downstream neighbor to the minigene construct significantly enhanced their excision (10). These findings suggest that interdependency between introns and splicing order are functionally linked. Two proposed models for this functional link are that *i)* efficient excision of local slowpoke introns is enhanced by *cis* elements in their neighboring introns or *ii)* their splicing is directly promoted by a neighboring act of splicing (10), but mechanistic evidence for each model is lacking.

Multiple 5′ and 3′ splice sites in the same pre-mRNA can compete for recognition by splicing factors and commitment to splicing, resulting in alternative splicing, a regulatory mechanism that plays critical roles in cellular identity and function (reviewed in (30)). Yet, how splice sites are recognized and paired within long multi-intronic pre-mRNAs is still poorly understood. The intron and exon definition models posit that splice sites are first paired across the shortest unit between them, either the intron or the exon (31, 32). In lower eukaryotes, where genes mostly consist of short introns (<250 nucleotides) separated by longer exons, intron definition is thought to prevail. By contrast, when long introns are separated by shorter exons, as is the case in most vertebrate genes, exon definition is predominant (32, 33). A recent study showed that cross-intron and cross-exon pre-B complexes are structurally and functionally equivalent, and suggested that interaction between the cross-exon pre-B complex and the U1 snRNP bound to an upstream 5′ splice site triggers the handover of this splice site from U1 snRNP to U6 snRNP, yielding a cross-intron B complex that is subsequently activated to catalyze excision of the intron (Fig. S1*A*) (34). Alternatively, the recently proposed “U1 relay model” posits that U1 snRNP and U2AF2 are recruited to the 5′ and 3′ splice sites of an intron co-transcriptionally and sequentially (35). The 5′ splice site of the downstream intron displaces U1 snRNP from the 5′ splice site of the upstream intron, which promotes the interaction of the upstream 5′ splice site with U6 snRNA and the subsequent transition from the pre-B to the B complex, while U1 snRNP is transferred to the downstream 5′ splice site (Fig. S1*B*) (35). Both models could explain why human genetic variants in splice sites often lead to exon skipping rather than intron retention (32, 35). However, both models require that commitment to splicing proceeds sequentially, such that the downstream 5′ splice site is still present when U2 snRNP is recruited to the upstream 3′ splice site and/or when U1 snRNP is displaced from the upstream 5′ splice site. However, this appears incompatible with the fact that splicing order does not proceed strictly in the direction of transcription, notably in the case of local slowpoke introns, some of which are dependent on prior excision of their downstream intron (10). A unifying model, which has yet to be tested, proposes that splice site commitment and splicing catalysis may be kinetically independent (2, 36). Splice site commitment would occur co-transcriptionally from 5′ to 3′, with recruitment of U1 and U2 snRNPs to the 5′ splice site and the branch point, followed by A complex assembly on the Pol II surface as each intron is transcribed. There may then be a substantial delay until splicing catalysis, which would be functionally independent from transcription, leading to more heterogeneous intron excision timings and delayed removal of certain introns relative to their neighbors (2, 36).

In this study, we developed a multi-intron minigene reporter assay to interrogate the mechanisms underlying the splicing dependency between neighboring introns. We identified *EIF4G2* intron 18 as a local slowpoke intron that is excised after its upstream and downstream neighbors in minigene and endogenous transcripts. Mutagenesis experiments revealed that efficient intron 18 excision is strongly dependent on the initial presence of a downstream intron. This effect is partially mediated through the 5′ splice site of its downstream intron, corroborating the role of exon definition in coordinating adjacent splicing reactions. Furthermore, efficient intron 18 excisions only occurs if its downstream intron is splicing-competent. Our findings support a model by which intron 18 splice site commitment occurs before its neighbors are removed, while its excision necessitates a prior downstream act of splicing, thereby bridging the gap between exon definition, splicing order, and interdependency in intron excision.

## Results

### *EIF4G2* intron 18 is always removed last in endogenous and minigene transcripts

**Figure 1:**
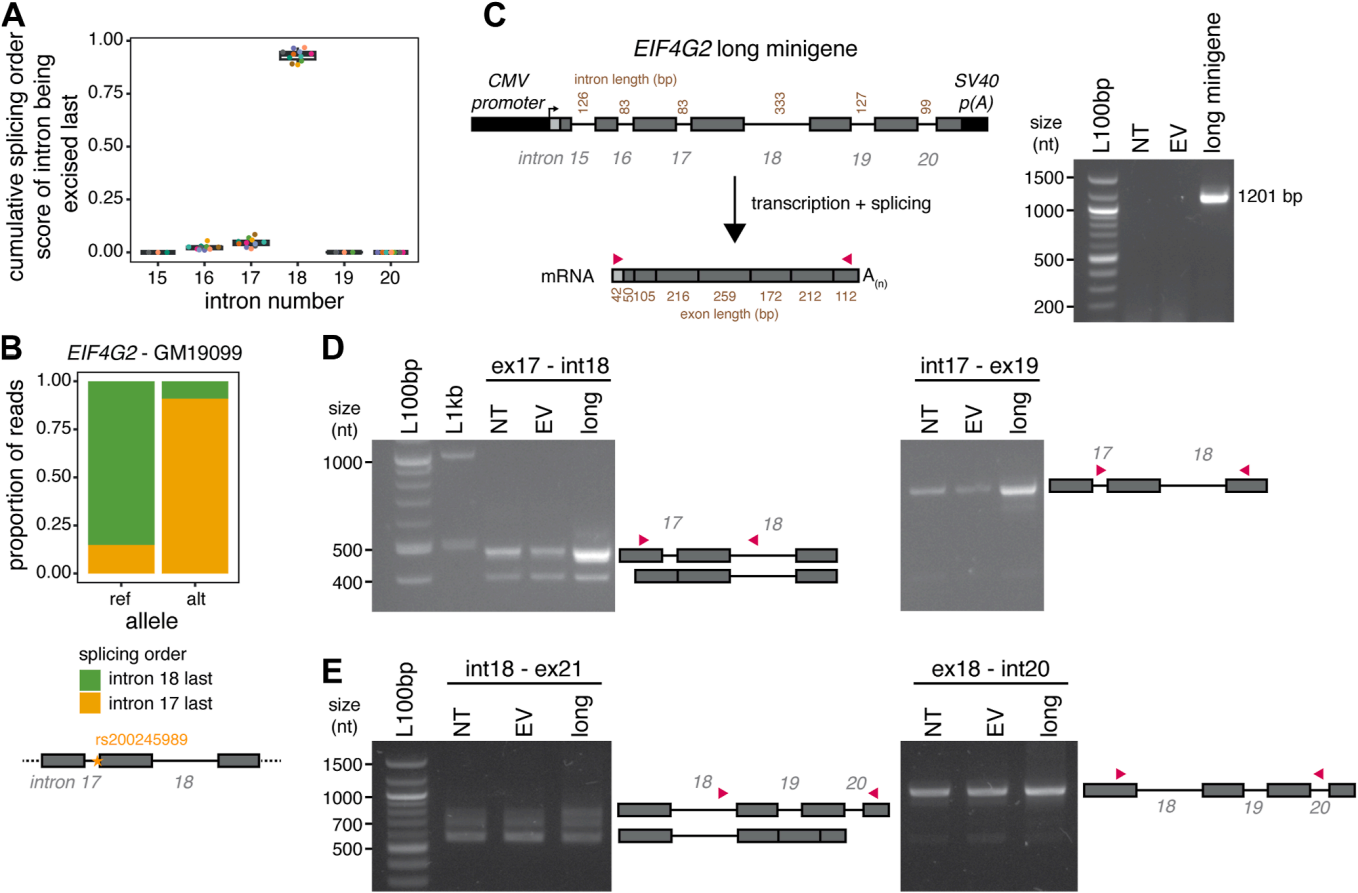
***EIF4G2* intron 18 is a slowpoke intron.***A,* frequency of each intron being excised last within *EIF4G2* introns 15 to 20, represented using the cumulative splicing order score for all splicing orders in which the given intron is excised last. Individual splicing orders and associated scores are shown in Table S1. Each *colored dot* represents a distinct human LCL. Intron 18 consistently has the highest score, indicating that it is almost always excised last in each LCL. *B,* allele-specific splicing order for introns 17 and 18 in LCL GM19099. “Ref” and “alt” refer to the genotypes for rs200245989. *C,left:* schematic representation of the *EIF4G2* long minigene and the resulting mRNA after expression and splicing in HEK293T cells. Exons are shown as *dark gray rectangles* and introns as *lines*. The *light gray rectangle* represents the plasmid sequence between the cytomegalovirus (CMV) promoter and the start of the *EIF4G2* sequence. The *dark pink arrowheads* indicate the PCR primers used for RT-PCR analysis of minigene transcripts. *Right*: Agarose gel electrophoresis of the minigene RT-PCR product. The forward primer is specific to the minigene. *D,* agarose gel electrophoresis of RT-PCR products for splicing order analysis of *EIF4G2* introns 17 and 18 with the two primer pairs shown as *dark pink arrowheads*. All products were migrated on the same gel, which was split in two for easier schematic representation of the obtained products. Note that primers are not minigene-specific and amplify the endogenous gene in NT and EV samples. *E,* same as D, but for splicing order analysis of introns 18–20. bp, base pair; EV, empty vector; EV, empty vector; L100 bp, 100 bp ladder; L1kb, 1 kb ladder; LCL, lymphoblastoid cell line; NT, no transfection.

To investigate the mechanisms underlying interdependency between local slowpoke introns and their neighbors, we searched for a model intron that is systematically excised after all other proximal introns. We mined our published dataset of splicing orders computed from direct chromatin-associated RNA nanopore sequencing from human lymphoblastoid cell lines (LCLs), HeLa cells, K562 cells, neuronal progenitor cells and motor neurons derived from human induced pluripotent stem cells (18, 29). We selected *EIF4G2* intron 18 (from RefSeq transcript NM_001418.3) because *i)* it was always removed last within the region spanning introns 15 to 20 across cell types (Figs. 1*A* and S2*A*, Table S1); and *ii)* we previously identified a splicing order reversal between introns 17 and 18 in LCL GM19099, which occurred together with a rare single nucleotide polymorphism (SNP, rs200245989) located three base pairs from the junction between intron 17 and exon 18 (Fig. 1*B*) (29). Overall, these results indicate that *EIF4G2* intron 18 is a *bona fide* local slowpoke intron whose splicing order can be modulated by a specific sequence change, thus representing an ideal model to investigate the underlying mechanisms.

Because slowpoke introns have been found to be dependent on either their upstream or downstream introns (10), we sought to study the mechanisms of *EIF4G2* splicing order in a multi-intronic context. We designed a multi-intron splicing reporter construct consisting of *EIF4G2* introns 15 to 20 (“long minigene”) under the control of the cytomegalovirus (CMV) promoter (Fig. 1*C*). After transfection in HEK293T cells for 24 h, we extracted total RNA and performed RT-PCR with a forward minigene-specific primer and a reverse primer targeting *EIF4G2* exon 21. We obtained a single band of 1201 base pairs, corresponding to the expected spliced product, indicating that all introns were efficiently excised from the minigene (Fig. 1*C*). Next, we asked whether splicing order was consistent between endogenous and minigene *EIF4G2* transcripts. Since minigene transcripts are not chromatin-associated, making it more challenging to isolate newly synthesized RNA, we used a previously described PCR-based strategy (25, 26) with exon/intron primer pairs to map the removal order of intron 18 and its neighbors (Fig. 1, *D* and *E*). For both endogenous and minigene transcripts, PCR with primers in exon 17 and intron 18 yielded two bands corresponding to the unspliced product and a product in which intron 17 had been removed (Fig. 1*D*, left panel). By contrast, PCR with primers in intron 17 and exon 19 yielded only the unspliced product (Fig. 1*D*, right panel). These results indicate that intron 17 is removed before intron 18. Similar experiments with primers in intron 18/exon 21 and exon 18/intron 20 revealed that introns 19 and 20 are excised before intron 18 (Fig. 1*E*). Thus, intron 18 is removed after its upstream and downstream neighbors in minigene transcripts, demonstrating that our multi-intron splicing reporter recapitulates the splicing order observed in endogenous transcripts.

### *EIF4G2* intron 18 excision requires the presence of downstream intron 19

**Figure 2:**
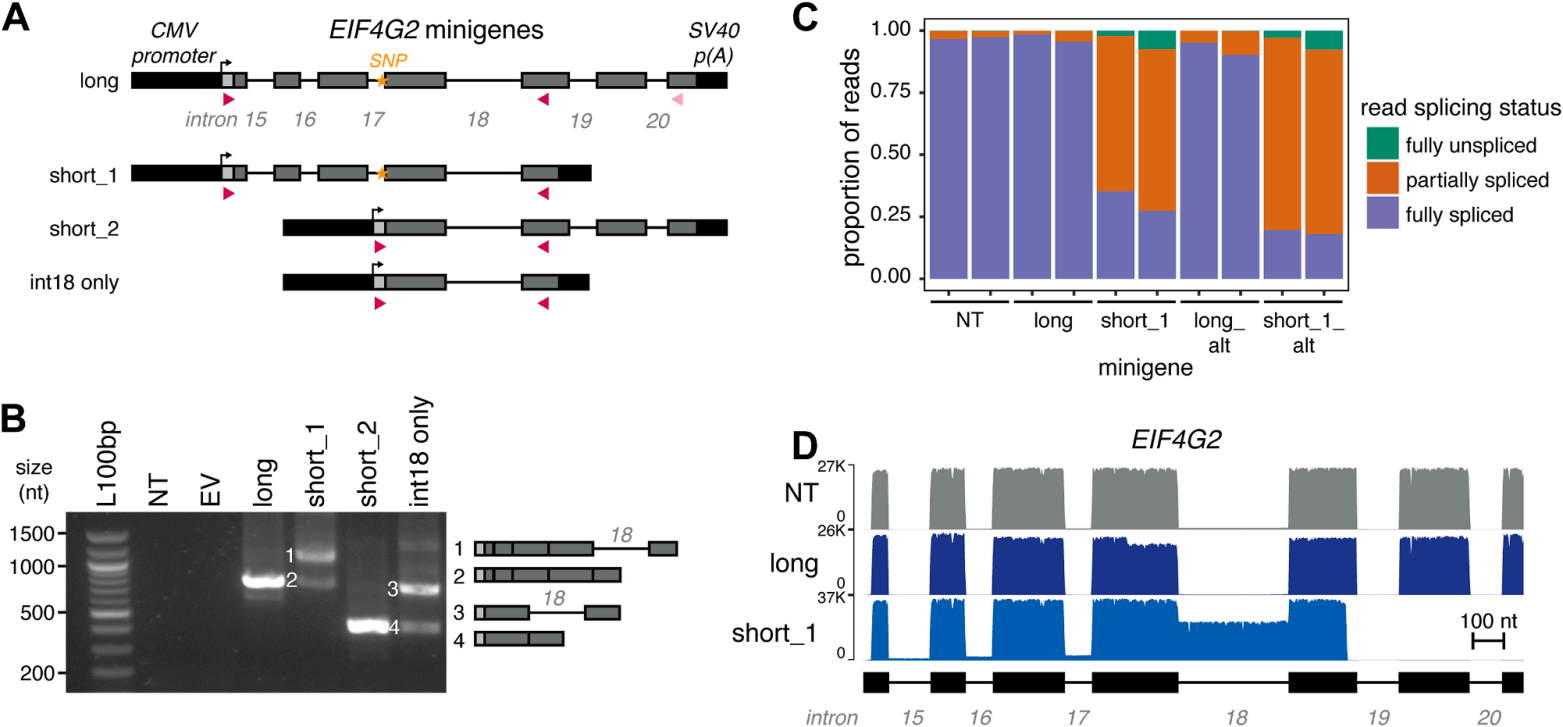
***EIF4G2* intron 18 excision depends on its downstream introns.***A,* schematic representation of the minigenes used in the other panels. Exons are shown as *dark gray rectangles* and introns as *lines*. The *light gray rectangle* represents the plasmid-specific 5′-UTR. The *orange star* represents single nucleotide polymorphism rs200245989 present in long_alt and short_1_alt minigenes. *B,* agarose gel electrophoresis of the RT-PCR products obtained from the expression of each minigene in HEK293T cells, using the primers shown as *dark pink arrowheads* in A. Each major product is numbered and schematized on the *right*. *C,* nanopore sequencing of RT-PCR amplicons obtained from minigene transcripts, showing the proportion of spliced, partially spliced and unspliced reads. For the long, short_1, long_alt and short_1_alt minigenes, a forward minigene-specific primer was used with a reverse primer in exon 19 (*dark pinkarrowhead* in A) or exon 21 (*light pinkarrowhead* in A), respectively. RT-PCR in NT was performed with primers in exons 15 and 21. Only reads spanning the full expected amplicons were included. Two biological replicates are shown for each condition. *D,* coverage tracks from nanopore sequencing of RT-PCR amplicons performed in *C*. bp, base pair; int, intron; K, thousands; L100 bp, 100 bp ladder; NT, no transfection.

We next asked whether excision of intron 18 is dependent on its upstream and/or downstream introns, as previously reported for other local slowpoke introns. We designed new minigenes consisting of introns 15 to 18 (“short_1”), introns 18 to 20 (“short_2”) and intron 18 alone (Fig. 2*A*). Transfection of the single intron minigene followed by RT-PCR resulted in two distinct bands corresponding to the spliced and unspliced transcripts (bands 3 and 4, Fig. 2*B*), with the latter being more pronounced, suggesting inefficient intron 18 excision. Similarly, absence of introns 19 and 20 in the short_1 minigene yielded an additional band of higher molecular weight compared to the long minigene, indicating an accumulation of incompletely spliced transcripts (band 1, Fig. 2*B*). Nanopore sequencing of RT-PCR amplicons revealed that most of these partially spliced transcripts showed intron 18 retention only (51–54% of reads), while a smaller proportion also displayed retention of upstream introns (9–19% of reads) (Fig. 2, *C* and *D*). To evaluate the impact of multi-intron minigene expression levels on intron 18 retention, we deleted nucleotides 1 to 441 of the CMV enhancer and promoter (37) in the short_1 and long minigenes (Fig. S2*B*). Despite a >4.5-fold reduction in steady-state minigene transcript levels (Fig. S2*C*; long minigene: 4.6-fold; short_1 minigene: 4.8-fold), intron 18 retention was consistently observed in the short_1 minigene (Fig. S2*B*), indicating that this splicing defect is not due to high minigene overexpression. Together, these results show that efficient intron 18 excision requires the initial presence of its downstream introns.

Turning our attention to the upstream region, we found that absence of introns 15 to 17 did not impair the excision of intron 18 in the short_2 minigene (Fig. 2*B*). We also cloned the alternative allele of GM19099, which carries the SNP rs200245989 (chr11:10800351T>C) in the 3′ splice site of intron 17, as long or short_1 minigenes (long_alt and short_1_alt, Fig. 2*A*) to determine whether delayed removal of intron 17 relative to intron 18 affected the excision of either intron. Splicing order analysis of the long_alt minigene showed increased levels of intron 17 being removed after intron 18 compared to the long minigene, as observed in GM19099 (Fig. S3*A*). Intron 18 retention was moderately increased in the short_1_alt minigene (78%) compared to the short_1 minigene (66%, Fig. S3*B*). However, the splicing order reversal did not impact splicing completion in the presence of downstream introns. Despite a slight increase in partially spliced reads due to modest intron 17 retention in the long_alt minigene (Fig. S3*C*), both long and long_alt minigenes were almost fully spliced (>90%, Fig. 2*C*), corroborating the independence between introns 17 and 18. Thus, we focused subsequent experiments on the connection between intron 18 and its downstream introns.

**Figure 3:**
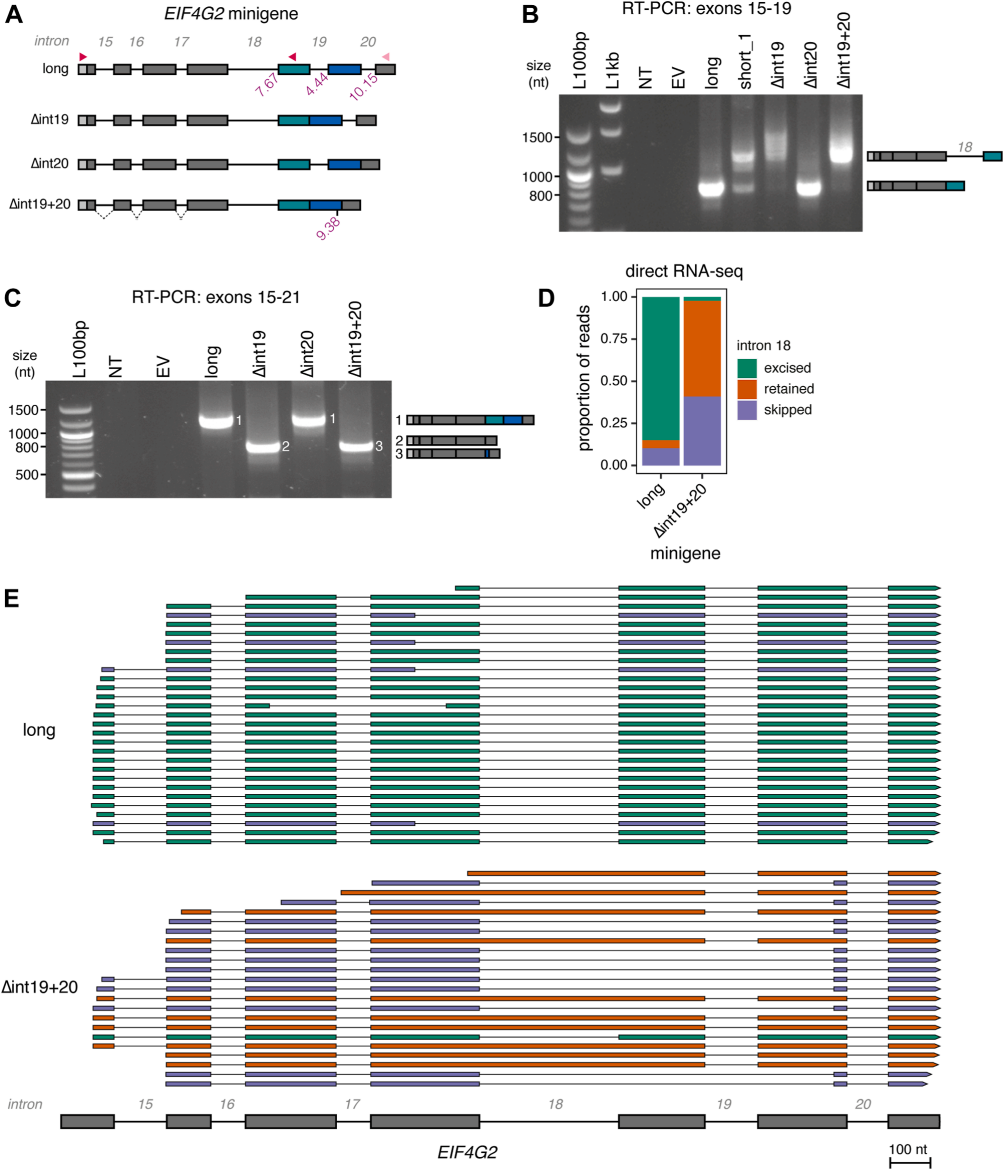
***EIF4G2* intron 18 excision depends on intron 19.***A,* schematic representation of the minigenes used in the other panels, with complete deletion of introns 19 and/or 20. *Exons* are shown as *dark gray rectangles* and introns as *lines*. The *light gray rectangle* represents the plasmid-specific 5′-UTR. The MaxEnt (68) 3′ splice site scores for the canonical 3′ splice sites of introns 18 to 20 (long minigene) and the cryptic splice site in exon 20 (Δint19+20 minigene) are shown in *purple font*. *B,* agarose gel electrophoresis of RT-PCR products obtained from the expression of each minigene in HEK293T cells, using the primers shown as *dark pink arrowheads* in A. Products are schematized on the *right*. *C,* same as *B*, but using the same minigene-specific forward primer and the reverse primer in exon 21 shown as *light pinkarrowhead* in A. The short_1 minigene was not included because it lacks exon 21. *D,* nanopore direct RNA sequencing (DRS) of minigene transcripts, showing the proportion of reads in which intron 18 is excised, retained or its 3′ splice site is skipped. Only reads corresponding to the complete minigene transcripts were included. *E,* representative reads from DRS of long and Δint19+20 minigenes. Each *arrow* represents one read, with *lines* indicating regions that were excised from the RNA. Displayed reads were randomly subsampled from the complete datasets and are colored according to the intron 18 splicing status as in D. bp, base pair; int, intron; L100 bp, 100 bp ladder; L1kb, 1 kb ladder.

To determine whether intron 18 excision depends on only one or both of its downstream introns, we used site-directed mutagenesis to delete intron 19 or intron 20 from the long minigene (Fig. 3*A*). While deletion of intron 20 had no detrimental impact on splicing (Fig. 3, *B* and *C*), deletion of intron 19 led to skipping of exons 19 and 20 (Fig. 3*C*). Consequently, although RT-PCR with a reverse primer in exon 19 detected partially spliced minigenes (Fig. 3*B*), these likely correspond to splicing intermediates that still contain exon 19, rather than intron retention. Intron 19 deletion results in a large composite exon (exons 19–20 together) that is > 400 nt and previous reports have shown that such expanded internal exons are often skipped in vertebrates (38). Thus, we cannot distinguish whether intron 18 is alternatively excised because of the absence of intron 19 *per se* or due to the resulting large flanking exon.

To circumvent this problem, we deleted both introns 19 and 20 (Δint19+20), resulting in the absence of internal exons downstream of intron 18. Minigene Δint19+20 behaved similarly to Δint19 alone, with retention of intron 18 when using a reverse primer in exon 19 (Fig. 3*B*). Curiously, when using a reverse primer in exon 21, the Δint19+20 minigene yielded a band of lower molecular weight compared to the long minigene (band #3, Fig. 3*C*), despite the absence of a canonical splice site downstream of intron 18 in the Δint19+20 minigene. To identify all minigene transcripts without potential biases from the selection of PCR primers, we performed nanopore direct RNA sequencing (DRS) on the long and Δint19+20 minigenes. In the Δint19+20 minigene, 57% of reads displayed intron 18 retention (Fig. 3*D*), indicating that PCR with the reverse primer in exon 21 significantly underestimated intron 18 retention, likely due to preferential amplification of a shorter cryptic splicing product. Indeed, 41% of DRS reads showed exclusion of exon 19 and most of exon 20, with the use of a cryptic 3′ splice site at position 181 of exon 20 (Fig. 3*E*). These observations indicate that the 3′ splice site of intron 18 cannot be used if intron 19 is absent from the initial pre-mRNA and that the presence of its downstream exons is not sufficient to promote correct intron 18 excision.

### The 5′ splice site of intron 19 contributes to efficient and accurate intron 18 excision

**Figure 4:**
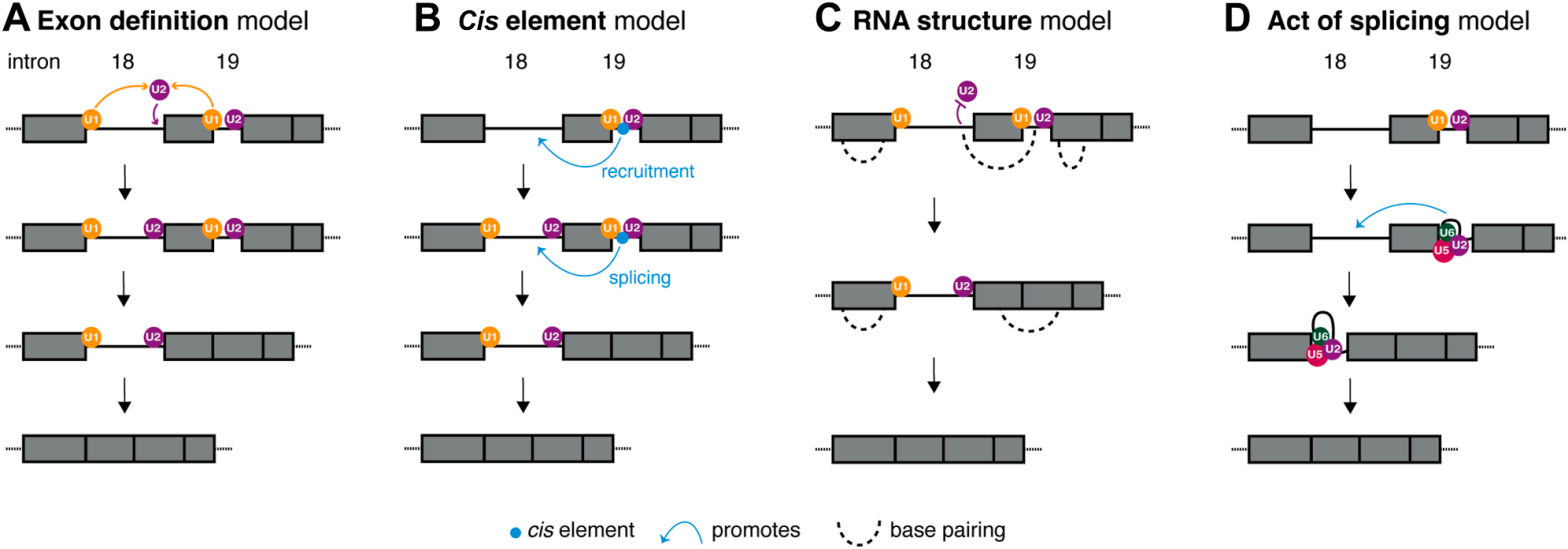
**Models for the interdependency between introns 18 and 19.***A,* in the exon definition model, binding of U1 snRNP to the downstream 5′ splice site enhances recruitment of U2 snRNP to the 3′ splice site of intron 18. *B,* in the *cis* element model, a regulatory sequence in intron 19 (distinct from the 5′ splice site) enables either recruitment of splicing factors to and/or excision of intron 18. *C,* in the RNA structure model, the presence of intron 19 results in a secondary structure that blocks binding of splicing factors to intron 18 splice sites or its branch point. Removal of intron 19 results in an alternative structure in which intron 18 is more accessible. *D,* in the act of splicing model, prior removal of intron 19 through splicing enables excision of intron 18.

Several mechanisms could explain the dependency of intron 18 excision on its downstream intron: 1) The 5′ splice site of intron 19 may be required for exon 19 definition and appropriate recruitment of the U2 snRNP to the intron 18 branch point (Fig. 4*A*); 2) There may be a *cis* element in intron 19 that contributes to efficient intron 18 excision (Fig. 4*B*); 3) A base pairing interaction between intron 18 and a downstream region may obstruct *cis* elements in intron 18 until intron 19 is removed, leading to a rearrangement of RNA secondary structure that favors binding of splicing factors to intron 18 (Fig. 4*C*). Indeed, structural elements involving splice sites or the branch point have been shown to impact splicing timing and/or efficiency (39, 40, 41); and 4) As proposed by Kim *et al.* (10), the act of removing intron 19 may promote intron 18 excision (Fig. 4*D*).

**Figure 5:**
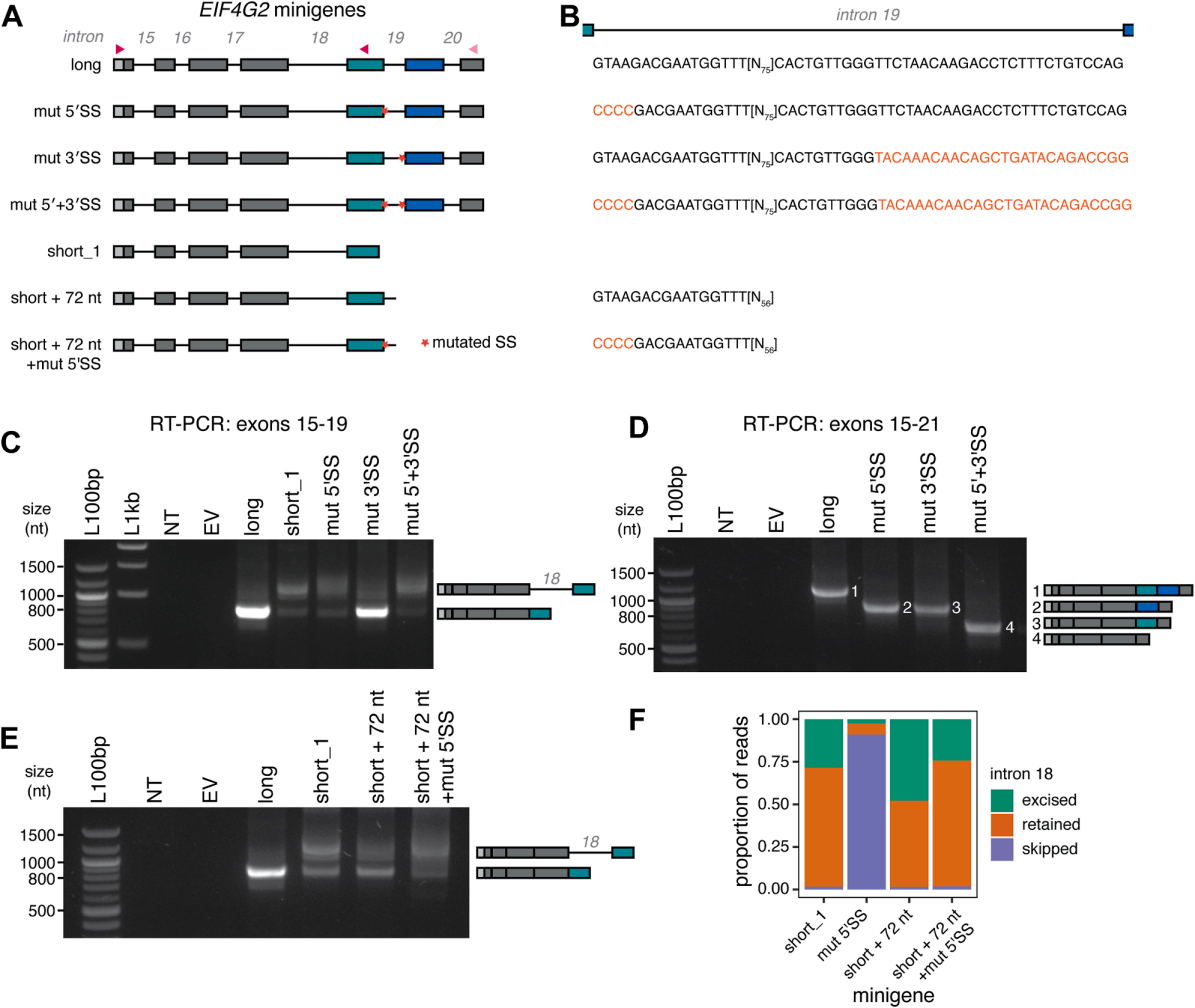
***EIF4G2* intron 18 excision partially depends on a functional downstream 5′ splice site.***A,* schematic representation of the minigenes used in the other panels, with 5′ and/or 3′ splice site mutations (*orange stars*). Exons are shown as *dark gray rectangles* and introns as *lines*. The *light gray rectangle* represents the plasmid-specific 5′-UTR. *B,* intron 18 sequence in each minigene from *A*, showing the mutated regions in *orange*. *C,* agarose gel electrophoresis of RT-PCR products obtained from the expression of the indicated minigenes in HEK293T cells, using the primers shown as *dark pink arrowheads* in A. Products are schematized on the *right.D,* Same as *C*, but using the same minigene-specific forward primer and the reverse primer in exon 21 shown as *light pinkarrowhead* in A. The short_1 minigene was not included because it lacks exon 21. Each product is numbered and schematized on the *right*. *E,* Same as C, but for the indicated minigenes. *F,* Nanopore direct RNA sequencing of the indicated minigene transcripts, showing the proportion of reads in which intron 18 is excised, retained or its 3′ splice site is skipped. Only reads corresponding to the complete minigene transcripts were included. SS: splice site; int: intron; mut: mutated; L100 bp: 100 bp ladder.

To test the contribution of exon definition to the dependency between introns 18 and 19 (model 1, Fig. 4*A*), we mutated the first four nucleotides (nt) of intron 19 in the long minigene (Fig. 5, *A* and *B*). We also mutated the last 26 nucleotides of intron 19, which was not expected to affect intron 18 excision if exon definition underlies its dependency on intron 19. Mutation of the 5′ splice site (mut 5′SS) yielded similar results to intron 19 deletion, with skipping of exon 19 (Fig. 5, *C* and *D*). Mutation of the intron 19 3′ splice site (mut 3′SS) led to exon 20 skipping but did not affect intron 18 excision, while mutation of both splice sites simultaneously caused skipping of exons 19 and 20 (Fig. 5, *C* and *D*). These observations are consistent with the exon definition model, whereby the 5′ splice site mutation prevents proper exon 19 definition. To validate the requirement of the downstream 5′ splice site for intron 18 excision, we added 72 nt of the 5′ portion of intron 19 to the short_1 minigene (short + 72 nt, Fig. 5*A*). Presence of this segment partially rescued intron 18 retention, but the effect was lost if the added 5′ splice site was mutated (short + 72 nt + mut 5′SS), as assessed by both RT-PCR and DRS (Fig. 5, *E* and *F*), supporting the model by which exon 19 definition is necessary for intron 18 excision.

**Figure 6:**
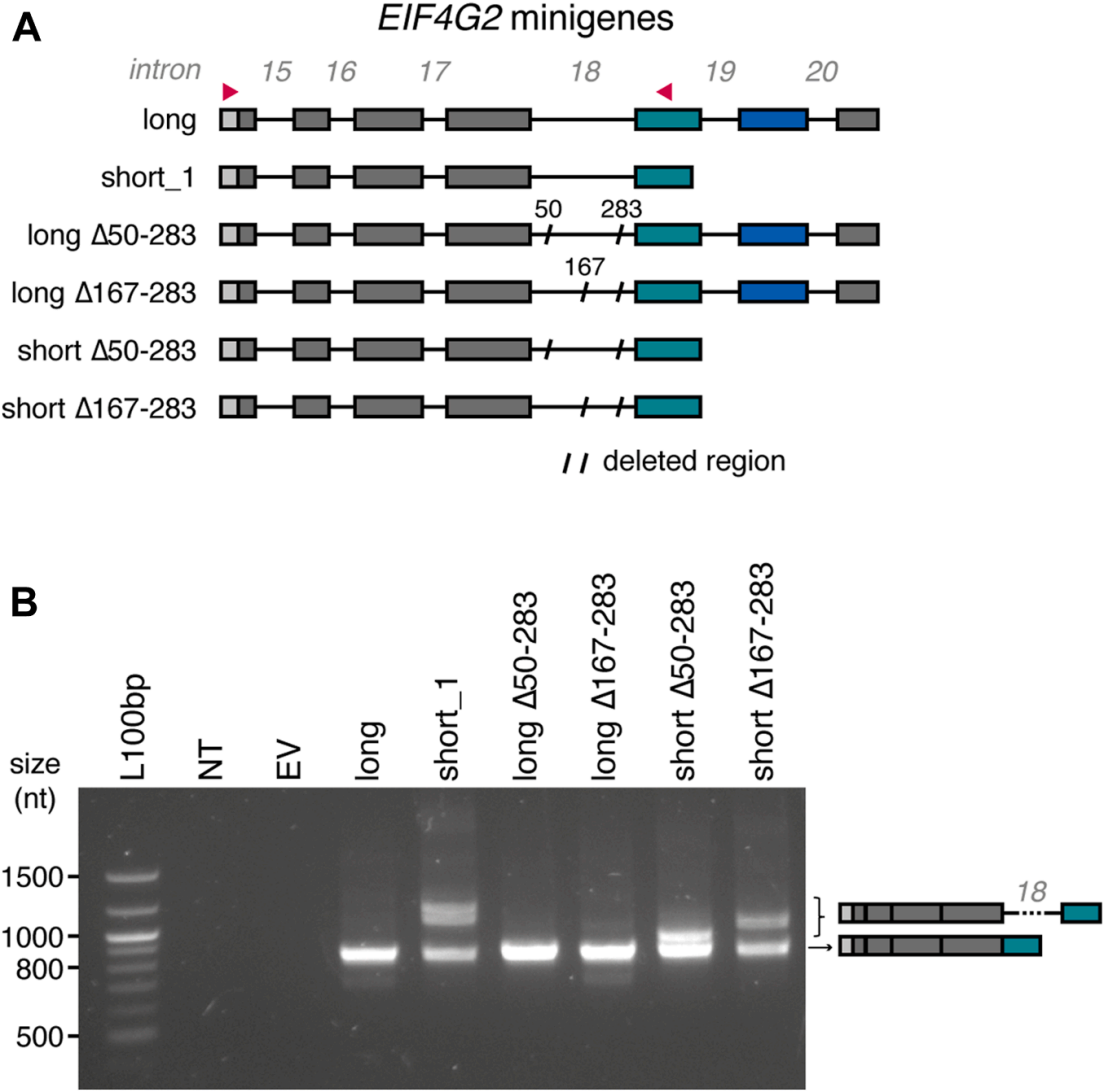
***EIF4G2* intron 18 dependency on its downstream intronis independent from its length.***A,* schematic representation of the minigenes used in B. Exons are shown as *dark gray rectangles* and introns as *lines*. The *light gray rectangle* represents the plasmid-specific 5′-UTR. Deleted regions in intron 18 are shown between slanted *vertical lines*, which are annotated with their position relative to the start of intron 18. *B,* agarose gel electrophoresis of RT-PCR products obtained from the expression of each minigene in *A* in HEK293T cells, using the primers shown as *dark pink arrowheads*. Products are schematized on the *right.* Amplicons with intron 18 retention (indicated by the curled bracket) vary in size due to the introduced deletions. bp, base pair; int, intron; L100 bp, 100 bp ladder.

Next, we investigated whether shortening intron 18 (333 nt) to <250 nt would switch its excision to a purely intron definition mode and alleviate its dependency on intron 19. We introduced two deletions in intron 18 (nucleotides 50–283 and 167–283) in the long and short_1 minigenes (Fig. 6*A*), resulting in lengths of 100 and 217 nt. The two flanking exons measured 259 and 172 nt and were either larger or similar in size to the deletion-containing intron 18, yielding an architecture compatible with intron definition. The long minigene transcripts with intron 18 deletion showed complete splicing, indicating that the deletions themselves did not impair intron 18 excision (Fig. 6*B*). However, neither deletion alleviated intron 18 retention in the short minigenes (Fig. 6*B*). These results suggest that the coupling between intron 18 and 19 excision is independent from the length of intron 18, and that intron 18 may be removed through exon definition even when it is shorter than its flanking exons.

Because intron 18 excision was not fully rescued by the addition of a downstream 5′ splice site in the short+72 nt minigene, we assessed the role of other potential intron 19 *cis* elements in promoting intron 18 removal (model 2, Fig. 4*B*). We randomly shuffled the internal sequence of intron 19 (positions 7–101) in the long minigene, leaving the 5′ splice site, 3′ splite site, and branch point intact. However, three distinct shuffling iterations had no impact on intron 18 excision (Fig. S3*D*), suggesting that the dependency between introns 18 and 19 is not mediated by internal intron 19 sequences.

**Figure 7:**
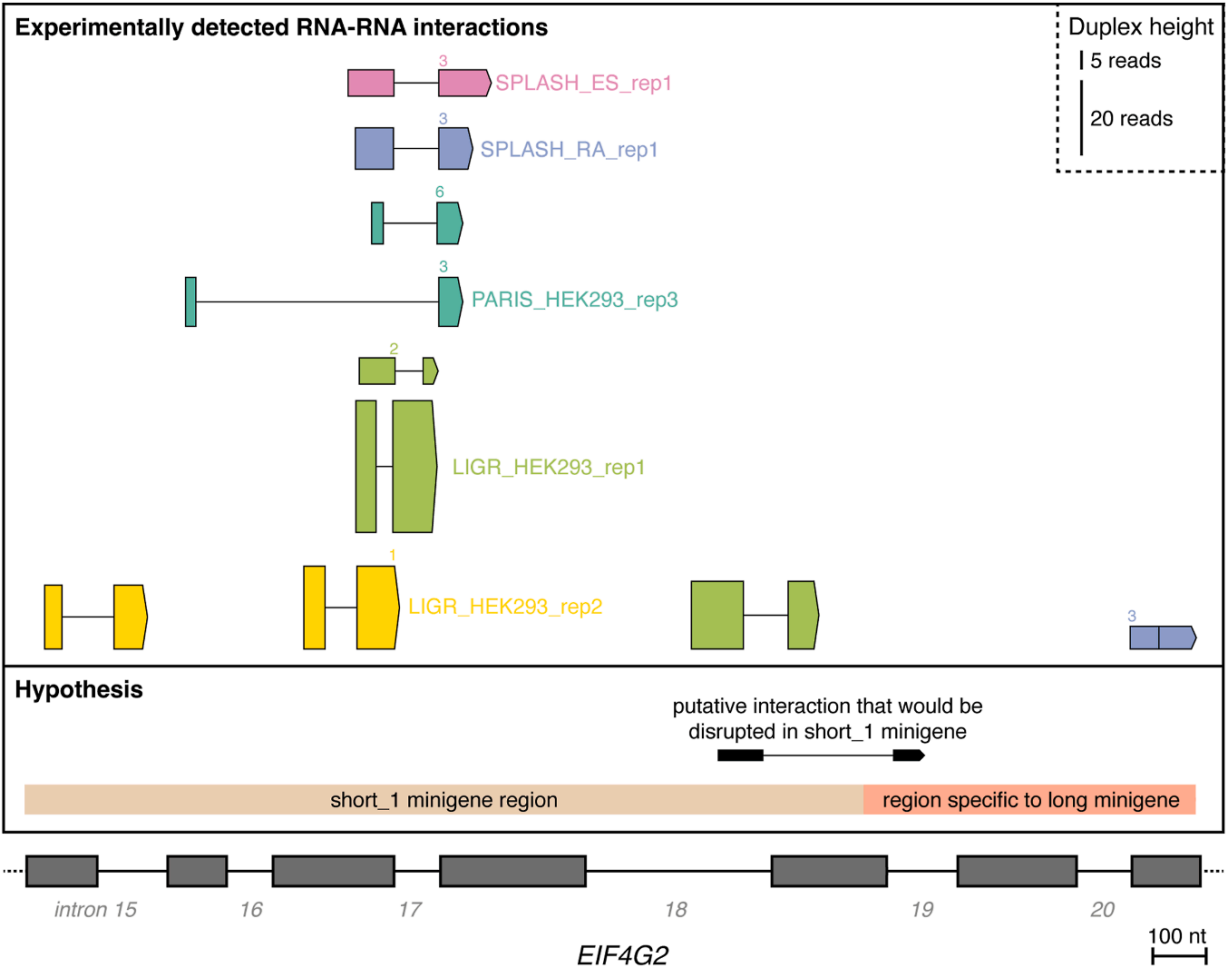
**RNA-RNA interactions in *EIF4G2*.***Top panel*: Duplexes identified in experimental high-throughput RNA-RNA interaction datasets (42, 43, 44). Each *arrow* represents one RNA-RNA duplex, with the two arms (*colored rectangles*) indicating the two RNA sequences interacting with one another. Only duplexes with at least one arm overlapping an intron by ≥ 1 nucleotide in the region corresponding to the *EIF4G2* long minigene are shown. The number of intronic nucleotides is indicated for breakpoints that are closer to splice junctions. The height of the duplex is proportional to the number of supporting reads, as shown in the top *right* legend. SPLASH, PARIS and LIGR-seq refer to the names of the methods used to map RNA-RNA interactions (42, 43, 44). ES: human embryonic stem cells; RA: human embryonic stem cells differentiated with retinoic acid for 5 days; rep: replicate. *Middle panel*: Schematic of the hypothesis in which an RNA-RNA interaction spanning intron 18 and the region specific to the long minigene would get disrupted in the short_1 minigene and contribute to intron 18 retention. *Bottom panel*: *EIF4G2* gene structure, with introns shown as *lines* and exons as *rectangles*.

We also investigated whether RNA-RNA interactions mediate intron 18 excision (model 3, Fig. 4*C*). If that were the case, we reasoned that these interactions likely involved base pairing between intron 18 and a region in *EIF4G2* that is only present in the long minigene (introns 19–20, exons 20–21), which would get disrupted in the short minigene (Fig. 7, middle panel). To explore this hypothesis, we analyzed published experimental high-throughput RNA-RNA interaction datasets obtained through crosslinking of RNA-RNA duplexes in living cells, followed by intramolecular proximity ligation and next-generation sequencing (42, 43, 44, 45, 46, 47, 48, 49). These datasets were collected from diverse human cell lines, including HEK293T cells, and were uniformly processed using our recently published tool DuplexDiscover (50). We identified 107 high-confidence RNA-RNA duplexes with both arms in *EIF4G2*, indicative of *cis* interactions, of which 35 were in the long minigene region (exons 15–21) (Table S2). To examine interactions that occurred in pre-mRNA, we filtered for duplexes with at least one arm overlapping an intron by at least one nucleotide. We found seven duplexes from five distinct datasets that showed a *cis* interaction between exons 17 and 18 and partially overlapped with intron 17 (Fig. 7), demonstrating our ability to uncover *cis* interactions involving introns in *EIF4G2* using this approach. Nevertheless, a single duplex with an arm in intron 18 was detected, with the second arm mapping to a region in exon 19 that is present in all minigenes (Fig. 7). Moreover, no interactions between intron 18 and regions specific to the long minigene were uncovered. Overall, our findings underscore the importance of proper exon 19 definition for accurate intron 18 excision, while internal intron 19 *cis* elements and secondary structure do not appear to play a major role.

### A downstream splicing event is required for efficient intron 18 excision

**Figure 8:**
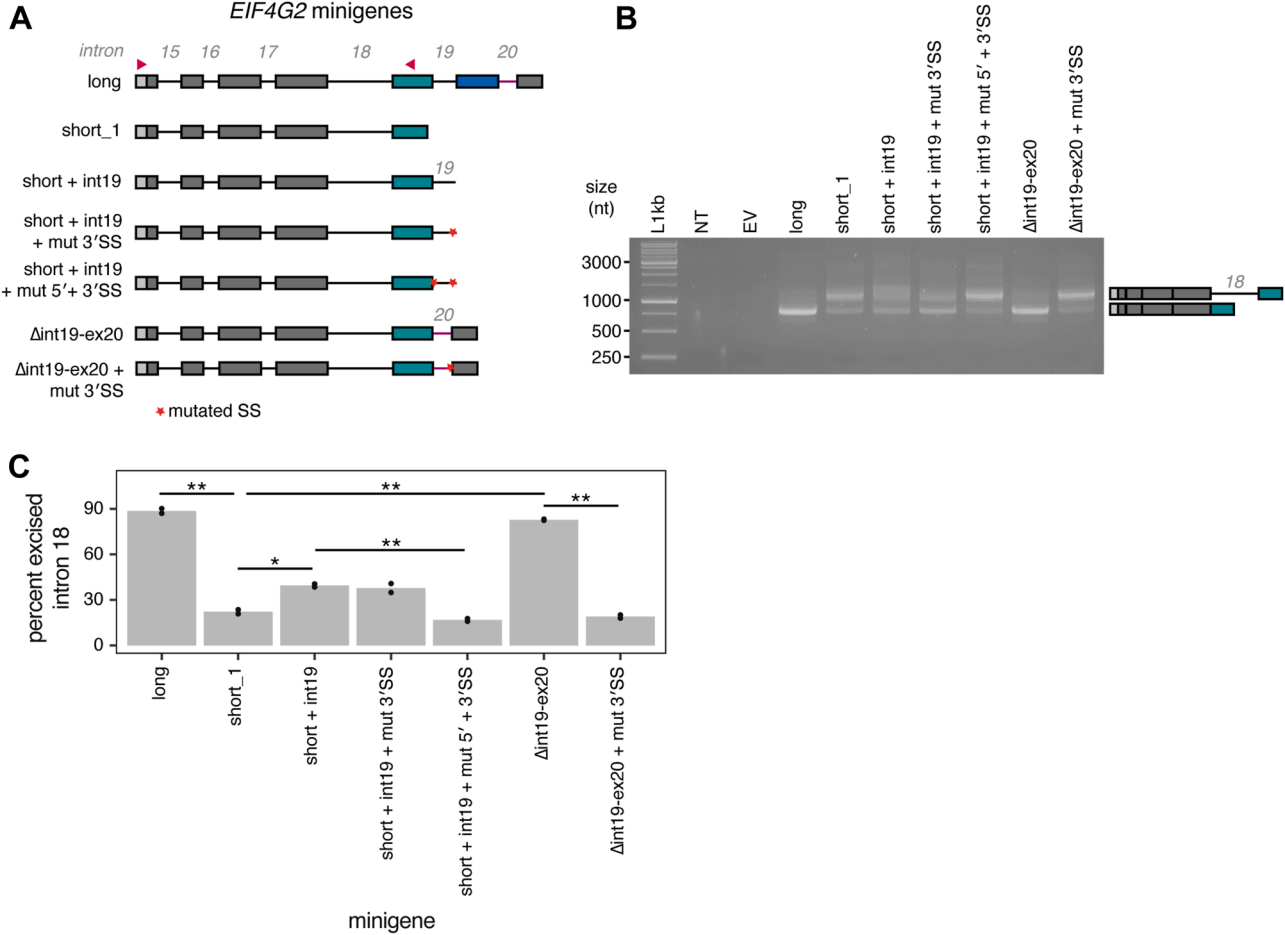
**Intron 18 excision requires a downstream act of splicing.***A,* schematic representation of the minigenes used in *B*. Exons are shown as *dark gray rectangles* and introns as *lines*. The *light gray rectangle* represents the plasmid-specific 5′-UTR. The *orangestars* indicate 5′ or 3′ mutated splice sites, as in Figure 5*B*. *B,* agarose gel electrophoresis of RT-PCR products obtained from the expression of the minigenes shown in A in HEK293T cells, using the primers shown as *dark pink arrowheads*. Products are schematized on the *right*. *C,* qRT-PCR of *EIF4G2* intron 18 excision levels in the indicated minigenes. Percent excised levels were calculated as 1/(1 + 2^ΔCt(spliced-unspliced)^). *Dots* represent two biological replicates from independent transfections. Minigenes were compared with an unpaired two-sided Student’s *t* test. ∗*p*-value < 0.05, ∗∗*p*-value < 0.01. SS: splice site; int: intron; mut: mutated; L1kb: 1 kb ladder.

The partial rescue of intron 18 excision in the short + 72 nt minigene implies that exon definition is not the only determinant of the interdependency between introns 18 and 19. Thus, we tested whether a downstream act of splicing is required for efficient intron 18 removal (model 4, Fig. 4*D*). First, we added the full intron 19 sequence to the short_1 minigene, but without a downstream exon (short + int19), reasoning that an incomplete 3′ splice junction would render intron 19 splicing-incompetent (Fig. 8*A*). We also generated the equivalent construct with a mutated 3′ splice site (Fig. 8*A*, short + int19 + mut 3′SS). RT-PCR with a reverse primer in the minigene-specific 3′ region confirmed that intron 19 was not excised in either construct (Fig. S3*E*). Consistent with the presence of an intact downstream 5′ splice site, these minigenes displayed intermediate levels of intron 18 retention compared to the short_1 minigene (Fig. 8, *B* and *C*). These results corroborate that the presence of intron 19 is not sufficient to promote intron 18 excision if it is not also splicing-competent. Moreover, mutating the 5′ splice site of intron 19 in the short + int19 + mut 3′SS construct (Fig. 8*A*) led to intron 18 retention levels comparable to the short_1 minigene (Figs. 8, *B*, *C* and S3*E*). This indicates that exon 19 definition (model 1) and the act of splicing intron 19 (model 4) are additive and are both required for complete removal of intron 18.

Lastly, we asked if any downstream intron could promote intron 18 excision or if intron 19 was specifically required. We deleted exon 20 from the Δint19 minigene (Δint19-ex20), such that intron 20 was immediately downstream of exon 19. Interestingly, intron 18 was efficiently removed in this minigene but this effect was abolished if the 3′ splice site of intron 20 was mutated (Fig. 8, *B* and *C*). Of note, some exon 19 skipping was observed in the Δint19-ex20 minigene (band #3, Fig. S3*F*), akin to the Δint19 minigene (band #2, Fig. 3*C*). However, intron 18 was efficiently excised when exon 19 was not skipped in the Δint19-ex20 minigene (band #2, Fig. S3*F*), contrary to the Δint19 minigene (Fig. 3*B*), potentially because of the large composite exon in Δint19 (exons 19–20 together). These results show that a functional downstream intron is the main requirement for efficient intron 18 excision, while the sequence identity of the downstream intron itself is not critical.

## Discussion

Several recent studies have highlighted the coordinated nature of splicing within mammalian pre-mRNAs (6, 10, 17, 18, 19, 20, 21, 22, 23), yet the underlying mechanisms remain mostly unknown. Here, we demonstrate that excision of a local slowpoke intron, defined as being removed after its neighbors, is dependent on its downstream intron and we provide evidence that this interdependency is mediated through the combinatorial effect of exon definition and a downstream act of splicing.

Although exon definition is thought to be the predominant mode of splicing in mammalian cells, transcriptome-wide studies have shown that numerous introns are removed after their downstream neighbors (10, 18, 22, 27, 28), raising the question of how exon definition occurs if the downstream 5′ splice site is no longer present. In two published examples of a mutation in a 5′ splice site impairing excision of the upstream intron, the upstream intron was removed prior to the downstream intron, meaning that the downstream 5′ splice site was still present and available for exon definition when the upstream intron was excised (17, 18). By contrast, the present study brings forth an example in which mutation of the downstream 5′ splice site impairs excision of the upstream intron even though the downstream intron is removed first. Given that the late intron 18 excision is observed across multiple cell types, these findings support the hypothesis that splice site commitment and intron excision are distinct steps that can be temporally separated. We propose that splice site recognition occurs in a coordinated manner on several neighboring introns when they are all still present (*e.g., EIF4G2* introns 18–20). In the exon definition model (33), after binding of U1 snRNP to each 5′ splice site, U2 snRNP is recruited to each 3′ splice site through the synergistic action of upstream and downstream U1 snRNPs (6) to form consecutive cross-exon A and then pre-B complexes (Fig. 4*A*), followed by conversion to cross-intron B complexes (34). Alternatively, in the U1 relay model (35), in which a single U1 snRNP is recycled for adjacent splicing reactions, spliceosome assembly proceeds from 5′ to 3′ until the cross-intron B complex is formed and U1 snRNP has been handed off to the next intron. In both models, after B complex formation, each intron is removed in a defined order (Fig. 4*A*), likely based on additional intron-specific regulatory signals. Interestingly, several proteins specific to activated B complexes are enriched on introns that are removed after their neighbors (22) and on detained introns (51), supporting that the pause between splice site commitment and intron excision occurs between the activated B complex and catalytically activated B complexes. This pause may enable fine-tuning of splicing timing and mRNA production in response to cellular needs.

Splicing order is largely conserved between cell types and is modulated by genetic variants, suggesting that it is primarily determined by nucleotide sequences (18, 27, 29). While our experiments show that intron 18 does not depend on specific sequences in intron 19, as its sequence can be shuffled or replaced by intron 20 (Figs. 8, *B*, *C* and S3*D*), regulatory motifs such as splicing enhancers or silencers may mediate delayed intron 18 removal. Previous analysis of binding motifs for 176 RNA-binding proteins (RBP) within intron pairs showed enrichment of a single motif, bound by the splicing factor SRSF3, in last-excised introns (10). Interestingly, addition of this motif further delayed excision of a local slowpoke intron, but had no impact when inserted in an early-excised intron (10), suggesting that multiple factors contribute to delayed intron removal and that these may be intron-specific. Furthermore, 16 RBP motifs were found enriched in first-excised introns, with the majority consisting of U-rich sequences (10). Consistently, U-rich sequences and U2AF2 binding were associated with early splicing in a separate study (28). Intron removal timing may also be mediated by RNA modifications. Indeed, a single intronic pseudouridine was sufficient to enhance splicing *in vitro*, and pseudouridines were enriched in binding sites of RBPs, including U2AF2 (52). N^6^-methyladenosine modifications were also found to regulate splicing in a context-dependent manner, including for detained introns (5, 53, 54), which are excised in some contexts but not others. In the future, DRS of nascent RNA could be used to interrogate the association between RNA modifications (55) and splicing order. Moreover, combining DRS with perturbation of RBPs, including splicing factors and RNA modification writers, readers and erasers, will help illuminate their role in splicing order determination.

Surprisingly, shortening intron 18 did not alleviate its dependency on intron 19, suggesting that intron 18 length – absolute and relative to neighboring exons – is not the only determinant of its excision through exon definition. A recent study demonstrated that the difference in GC content between introns and their flanking exons is a key discriminator between intron and exon definition (11). The differential architecture is characterized by longer introns with low GC content (≤40%) and relatively higher exon GC content, while the leveled architecture is composed of shorter introns with high GC content flanked by exons with similarly high GC content (11). Shortening introns in minigenes with differential architecture did not affect splicing (11), akin to the results obtained here for *EIF4G2*. Indeed, with respective GC contents of 42, 36 and 37%, *EIF4G2* exon 18, intron 18 and exon 19 are more consistent with the differential architecture, which was maintained with the shortened versions of intron 18 (GC contents of 31–33%). Nevertheless, the gene *IFRD2*, in which we previously observed similar dependency of an upstream intron on its downstream 5′ splice site for efficient excision (18), suggestive of exon definition, is characterized by a leveled architecture. Thus, additional properties intrinsic to each intron may contribute to removal through intron or exon definition. For instance, co-transcriptional lariat sequencing data indicate that many human introns can be excised independently from the downstream 5′ splice site, presumably through intron definition, and that this is favored by a strong polypyrimidine tract enabling optimal U2AF2 binding (28). Future studies will allow determining whether other local slowpoke introns also depend on exon definition.

Importantly, exon definition was not the only requirement for interdependency in intron excision, as our data indicates that adding the 5′ splice site of intron 19 or a splicing-incompetent intron 19 to the short_1 minigene is not sufficient to fully alleviate intron 18 retention. In fact, other local slowpoke introns were found to be dependent on their upstream intron for their removal (10), which cannot be explained by exon definition and synergistic U2 snRNP recruitment, as observed for *EIF4G2* intron 18. Our observations that efficient intron 18 excision can occur with a different downstream intron, as long as it is splicing-competent, suggest that local slowpoke introns depend on neighboring acts of splicing rather than specific *cis* elements in their flanking introns. Nevertheless, there appears to be a directional effect that may be specific to each slowpoke intron, as upstream prior splicing events had no impact on *EIF4G2* intron 18 retention. Interestingly, it was recently shown that the exon junction complex (EJC) regulates the coordinated inclusion of blocks of adjacent constitutive exons in a splicing order-dependent manner, with introns separating block exons excised first, while introns flanking the block are removed later (56). The authors postulated that EJC deposition on spliced block exons helped the recognition of flanking weaker splice sites, as the block exons were skipped in the absence of EJC components. These findings argue against a purely 5′ to 3′ recognition of splice sites, as proposed in the exon definition and U1 relay models. While different models may apply to distinct introns, EJC deposition may also help to promote the excision of later-excised introns that are paused between splice site commitment and splicing catalysis. Such a model could explain why skipping of exons 19 to 20 is favored over intron 18 excision in the Δint19 *EIF4G2* minigene, since the EJC would not be deposited near the junction between exons 19 and 20. Another possibility is that some splicing factors (*e.g.* required for catalytic activation or splicing catalysis) are recruited to intron 18 only if they can be recycled after completing a nearby splicing reaction. However, why such a mechanism would only affect certain introns in a directional manner remains unknown. In the future, experiments that allow tracking splicing factors and their association with RNA over time may provide further insights into the mechanisms underlying interdependency between local slowpoke introns and their neighbors.

Our findings also have important implications for the more than 15% of disease-causing genetic variants that are in splice sites or splicing regulatory elements (57). Two-intron minigene assays are frequently used to determine whether and how such variants disrupt pre-mRNA splicing (58, 59, 60). Yet, recent studies have shown that the surrounding sequence context influences how genetic variants or other perturbations impact splicing, including in minigene assays (61, 62, 63, 64). Our findings provide further evidence that some introns are not correctly excised in the absence of their neighbors. Thus, human genetics studies should prioritize minigenes with larger genomic context when feasible, including splicing-competent neighboring introns, as this is more likely to faithfully recapitulate splicing of endogenous transcripts and to accurately reveal how variants impact pre-mRNA splicing in genetic disorders.

## Experimental procedures

### Splicing order analysis of endogenous *EIF4G2* transcripts

*EIF4G2* splicing order scores were computed by multiplying the frequency of intermediate isoforms required to proceed through a given splicing order, as previously described (18, 29), using published DRS data from LCLs, K562 cells, HeLa cells, and induced pluripotent stem cells-derived neuron progenitors and motor neurons (GEO accession numbers GSE256190, GSE208225, GSE232455). Splicing order scores are bound by 0 and 1, with the most frequent splicing orders have higher scores. In all LCLs except GM19099, splicing order was analyzed simultaneously for introns 15 to 20 (Fig. 1*A*, Table S1). Due to the lower sequencing depth in datasets from other cell types, splicing order that had previously been separately computed for introns 16 to 18 and 18 to 20 was extracted from Table S5 of reference (18) (Fig. S2*A*). The cumulative splicing order score for each intron being excised last (Figs. 1*A* and S2*A*) was calculated by summing scores from all splicing orders in which the considered intron was excised last. For allele-specific splicing order analysis in GM19099, reads were mapped to their parental allele of origin and splicing statuses were computed as previously described (29). We extracted splicing statuses from all reads spanning introns 17 and 18 and filtered for reads in which one intron was excised and one intron was still present. The frequency of intron 18 or intron 17 being still present in the read (excised last) was computed for each allele (Fig. 1*B*).

### Cloning of *EIF4G2* minigenes

Genomic DNA was extracted from LCL GM19099 (Corriell) using DNeasy Blood & Tissue kit (Qiagen, cat. no. 69504). *EIF4G2* regions spanning exons 15 to 19 and exons 15 to 21 were amplified from genomic DNA using primers with MfeI and XhoI restriction sites (Table S3) and the Phusion High-Fidelity PCR Master Mix [New England Biolabs (NEB), cat. no. M0531] according to the manufacturer’s instructions. PCR products were run on an agarose gel for 1 h at 130V. Bands of the expected size were purified using the MinElute Gel Extraction kit (Qiagen, cat. no. 28604). PCR products or 5 μg of plasmid pCMV-M1 (Addgene, cat. no. 23007) were digested with 1 μl of MfeI and 1 μl of XhoI at 37 °C for 1 h. The linearized plasmid was run on an agarose gel for 1 h at 130V and purified using the MinElute Gel Extraction kit. Digested PCR products were purified using the Monarch PCR & DNA Cleanup kit (NEB, cat. no. T1030). Ligation was performed with 7 μl of purified PCR product, 50 ng of digested plasmid, 1 μl of T4 DNA Ligase (NEB, cat. no. M0202S) and 1 μl of T4 DNA Ligase buffer in a total volume of 10 1 μl. Ligation reactions were incubated at room temperature for 1 h, transformed into 5-alpha competent *Escherichia* *coli* (NEB, cat. no. C2987), plated on LB-agar plates with 50 mg/ml of kanamycin and incubated at 37 °C overnight. Positive colonies were picked and individually grown in LB-agar with 50 mg/ml kanamycin. Plasmid DNA was extracted using the Monarch Plasmid Miniprep kit (NEB, cat. no. T1010) or the Plasmid Plus Midi Kit (Qiagen, cat. no. 12943). Full plasmid sequences were verified by nanopore sequencing through Primordium or the RNomics Platform at Université de Sherbrooke. Plasmids corresponding to the GM19099 reference allele (genotype A at SNP rs200245989) were used for all experiments (long and short_1, unless otherwise specified (long_alt and short_1_alt carrying genotype G at SNP rs200245989, Figs. 2*C* and S2*A*). Deletions and mutations were introduced using the Q5 site-directed mutagenesis kit (NEB, cat. no. E0554) according to manufacturer’s instructions. Primers used for mutagenesis are listed in Table S3. 5′ and 3′ splice site mutated sequences were adapted from (6).

### Cell culture

HEK293T cells (kind gift from François Bachand, Université de Sherbrooke) were grown in DMEM (Wisent, cat. no. 319-005-CL) supplemented with 10% fetal bovine serum and 1% penicillin-streptomycin and tested negative for *mycoplasma*. For minigene transfections, 0.6 million cells were plated in each well of a 6-well plate. Twenty-four hours later, cells were transfected with 2.5 μg of plasmid using 7.5 μl of Lipofectamine 3000 (Life Technologies, cat. no. L0000008). Cells were harvested 24 hours after transfection. All transfection and subsequent experiments were performed in ≥2 biological replicates.

### RNA extraction

Cells were lysed in 1 ml of QIAzol lysis reagent (Qiagen, cat. no. 79306) and incubated at room temperature for 5 min. After addition of 200 μl of chloroform, samples were vigorously mixed by hand for 15 s, incubated at room temperature for 2 to 3 min and centrifuged at 12,000×*g* for 15 min at 4 °C. The aqueous phase was transferred to a new tube, mixed with 500 μl of isopropanol and 2 μl of GlycoBlue (Thermo Fisher Scientific, cat. no. AM9515) and incubated for 10 min at room temperature. Samples were centrifuged at 12,000×*g* for 10 min at 4 °C. RNA pellets were washed twice by adding 1 ml of 75% ethanol and centrifuging at 7000×*g* for 5 min at 4 °C. Air-dried pellets were resuspended in 30 to 50 μl of nuclease-free water.

### DNase treatment and RT-PCR

DNase treatment was performed on 2 μg of RNA using amplification-grade DNase I (Thermo Fisher Scientific cat. no. 18068015) according to the manufacturer’s instructions. For reverse transcription, 700 ng of DNase-treated RNA was mixed with 2 μl of Random Primer Mix (60 μM, NEB cat. no. S1330) and 1 μl of 10 mM dNTPs in a total volume of 10 μl. After incubation at 65 °C for 5 min, 5 μl of 5X Induro RT reaction buffer, 0.2 μl of murine RNase Inhibitor (40 U/μl, NEB cat. no. M0314), 1 μl of Induro Reverse Transcriptase (200 U/μl, NEB, cat. no. M0681) and 4.8 μl of nuclease-free water were added to the samples. Reverse transcription was carried out by incubating at 25 °C for 2 min, 55 °C for 30 min and 95 °C for 1 min. PCR was performed with 1 μl of cDNA and the Q5 Hot Start High-Fidelity DNA Polymerase (NEB, cat. no. M0491) in 25 μl reactions according to the manufacturer’s instructions, including use of the High GC enhancer. Primers used for RT-PCR are listed in Table S4. The following cycling conditions were used: 98 °C for 30 s, 35 cycles of 98 °C for 10 s, 60 °C for 30 s and 72 °C for 1 min, 72 °C for 2 min. For agarose gel electrophoresis, 10 μl of the PCR reactions were mixed with 2 μl of 6X gel loading dye (NEB, cat. no. B7024S) and migrated on a 1% agarose gel for 45 min at 120V, along with 100 bp and/or 1 kb DNA Ladders (NEB, cat. no. N3231L and N3232L or FroggaBio, cat. no. DM010). qRT-PCR to measure *EIF4G2* expression levels (Fig. S2*C*) was performed by combining 3 μl of cDNA diluted in water at a ratio of 1:250, 10 μl of Luna Universal qPCR master mix (NEB, cat. no. M3003) and 0.5 μl of forward and reverse primers (10 μM, Table S4) in a total volume of 20 μl with the following cycling conditions: 95 °C for 30 s, 40 cycles of 95 °C for 5 s, 60 °C for 10 s, and a final melt curve (65 °C to 95 °C with 0.5 °C increment for 5 s). qRT-PCR to measure intron 18 excision levels (Fig. 8*C*) was carried out as described above, but a minigene-specific reverse transcription was performed with 1.2 μl of a reverse primer targeting the SV40 sequence (100 μM, Table S4) instead of the Random Primer Mix. The resulting cDNA was diluted at a ratio of 1:100 and 1.5 μl was combined with 5 μl of Luna Universal qPCR master mix and 0.25 μl of forward and reverse primers (10 μM, Table S4) in a total volume of 10 μl with the following cycling conditions: 95 °C for 30 s, 40 cycles of 95 °C for 5 s, 58 °C for 10 s, and a final melt curve (65 °C to 95 °C with 0.5 °C increment for 5 s). Two primer pairs were used, which respectively targeted the unspliced intron 18-exon 19 junction and the spliced exon 18-exon 19 junction. Intron 18 percent excised levels were calculated as 1/(1 + 2^ΔCt(spliced-unspliced)^), as previously done (65).

### Nanopore sequencing of RT-PCR amplicons

PCR was performed with 1 μl of cDNA as described above, but with only 20 cycles. For the endogenous transcripts and the long minigene, a reverse primer in exon 21 was used (Table S4). For the short minigene, a reverse primer in exon 19 was used (Table S4). PCR reactions were cleaned-up using the Monarch PCR and DNA clean-up kit (NEB, cat. no. T1030S). 10 to 130 ng of DNA from each sample was carried forward for end-repair using the NEBNext Ultra II End repair/dA-tailing Module (NEB, cat. no. E7546S). The DNA was mixed with 1X volume of AMPure XP beads (Beckman Coulter, cat. no. A63881), incubated on a rotator for 5 min at room temperature, washed twice with 80% ethanol and eluted in 10 μl water. For ligation of unique sample barcodes, 10 ng of end-prepped amplicon in a total volume of 7.5 μl was combined with 2.5 μl native barcode [Oxford Nanopore Technologies (ONT), cat. no. SQK-NBD114.24] and 10 μl of Blunt/TA Ligase Master Mix (NEB, cat. no. M0367). The ligation reaction was incubated for 20 min at room temperature and inactivated with EDTA. All samples were pooled together and mixed with 0.4X volume of AMPure XP beads. The pooled sample was incubated on a rotator for 10 min at room temperature, washed twice with 80% ethanol and eluted in 35 μl water. 30 μl was combined with 5 μl of native adapter (ONT, cat. no. SQK-NBD114.24), 10 μl of NEBNext Quick Ligation Reaction Buffer and 5 μl of Quick T4 Ligase (NEBNext Quick Ligation Module, NEB, cat. no. E6056). The ligation reaction was incubated for 20 min at room temperature, mixed with 0.4X volume of AMPure XP beads and incubated on a rotator for 10 min at room temperature. Sample clean-up and loading onto a FLO-MIN114 flow cell was performed with the Native Barcoding Kit 24 V14 (ONT, cat. no. SQK-NBD114.24) according to manufacturer’s instructions. Sequencing was performed on a MinION device.

### Direct RNA nanopore sequencing

DRS library preparation was performed using the kit SQK-RNA004 (ONT) with 1 μg of total RNA according to manufacturer’s instructions. Sequencing was performed on a FLO-PRO004RA flow cell using a MinION device. Sequencing was carried out until >130,000 reads were obtained, which was sufficient to analyze minigene splicing given its high overexpression level (Fig. S2*C*). Each flow cell was used three times. Between each sample, the flow cell was washed with the kit EXP-WSH004 (ONT) according to manufacturer’s instructions.

### Nanopore sequencing data processing and analysis

For cDNA-PCR sequencing, basecalling and demultiplexing were performed with guppy v6.5.7 (ONT). All reads that passed basecalling were aligned to the reference human genome GRCh38 using minimap2 v2.28 (66) with parameters -ax splice. For DRS, fast basecalling was performed during sequencing with MinKNOW or after sequencing with Dorado v0.7.3 (ONT) and model rna004_130bps_fast@v5.0.0. All reads that passed basecalling were aligned to the reference human genome using minimap2 v2.28 (66) with parameters -ax splice -uf -k14. Uniquely mapped reads were extracted as detailed in (65). Coverage tracks in Figures 2*D* and 3*E* were generated with pyGenomeTracks (67). Intron excision statuses (excised, retained or skipped) were determined using the alignment CIGAR strings as previously described (18, 65). For cDNA-PCR, only reads spanning all expected amplicon introns (*i.e.* 15–20 for no transfection control and the long minigene, introns 15–18 for the short_1 minigene) were kept in the analysis. For DRS, only reads spanning all expected minigene introns (*i.e.* introns 15–20 for the long, Δint19+20 and mut 5′SS minigenes, introns 15–18 for the short_1, short + 72 nt and short + 72 nt + mut 5′SS minigenes) were considered in order to exclude endogenous *EIF4G2* transcripts.

### Analysis of RNA-RNA interaction datasets

Previously published high-throughput RNA-RNA interactions datasets (42, 43, 44, 45, 46, 47, 48, 49) were processed using DuplexDiscoverer (50) to identify duplexes in *EIF4G2*. First, we compiled all publicly available datasets of RNA–RNA interaction profiling experiments, including RIC-seq, SPLASH, PARIS, LIGR-seq, PARIS2, KARR-seq, and CAR-SPLASH (42, 43, 44, 45, 46, 47, 48, 49). For each sample, we ran DuplexDiscoverer using parameters in accordance with the processing guidelines described in the original publications. High-confidence duplexes with more than five supporting reads were subsequently filtered for hypothetical RNA-RNA interactions to include only those involving *cis* interactions within the minigene region (exons 15–21 and intervening introns), for which at least one anchor spanned an intron by ≥ 1 nucleotides. Resulting duplexes were converted to BED12 format, with each anchor representing one block, and plotted using pyGenomeTracks (67).

## Data availability

The *EIF4G2* nanopore sequencing data generated in this article is available at the Gene Expression Omnibus (accession number GSE324595).

## Supporting information

This article contains supporting information (34, 35, 37, 42, 43, 44, 45, 46, 47, 48, 49).

## Conflict of interest

The authors declare that they have no conflicts of interest with the contents of this article.
