# Supplementary material for: Splicing dependency between EIF4G2 introns is mediated by exon definition and relies on a downstream splicing event
Source: J Biol Chem. 2026 Jun 27;302(8):113304. doi: 10.1016/j.jbc.2026.113304 (PMC13444286; doi:10.1016/j.jbc.2026.113304)
Supplement: Supplementary file 1 [file mmc1.pdf]

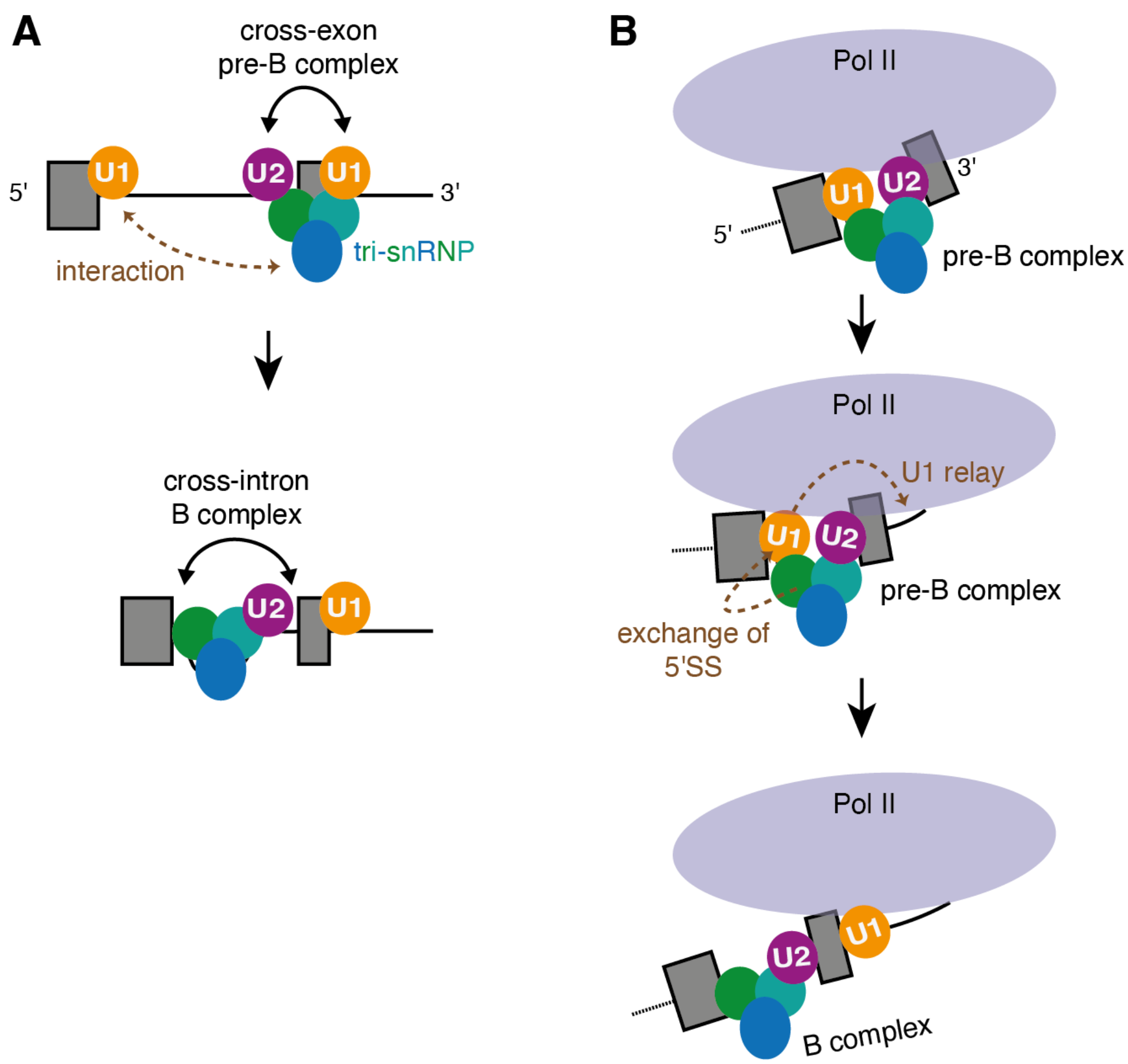

Figure S1. Simplified schematics of existing models for splice site recognition and pairing in human introns. A) In the exon definition model, splice sites are first recognized across exons through binding of U1 and U2 snRNPs to the 5′ and 3′ splice sites, respectively. According to the model in Zhang *et al.* (34), spliceosome assembly proceeds across the exon until the pre-B complex step, and transition to a cross-intron spliceosome occurs during the transition to the B complex, when the U1 snRNP at the upstream 5′ splice site (5′SS) is replaced by the U6 snRNP (shown in green within the tri-snRNP). B) In the U1 relay model described by Yoon *et al.* (35), binding of U1 and U2 snRNP to splice sites occurs co-transcriptionally and from 5′ to 3′ through physical interactions with RNA polymerase II (Pol II). Exchange of the upstream 5′SS between U1 snRNP and U6 snRNP allows recruitment of the same U1 snRNP to the downstream 5′SS (U1 relay). Schematics are adapted from (34, 35).

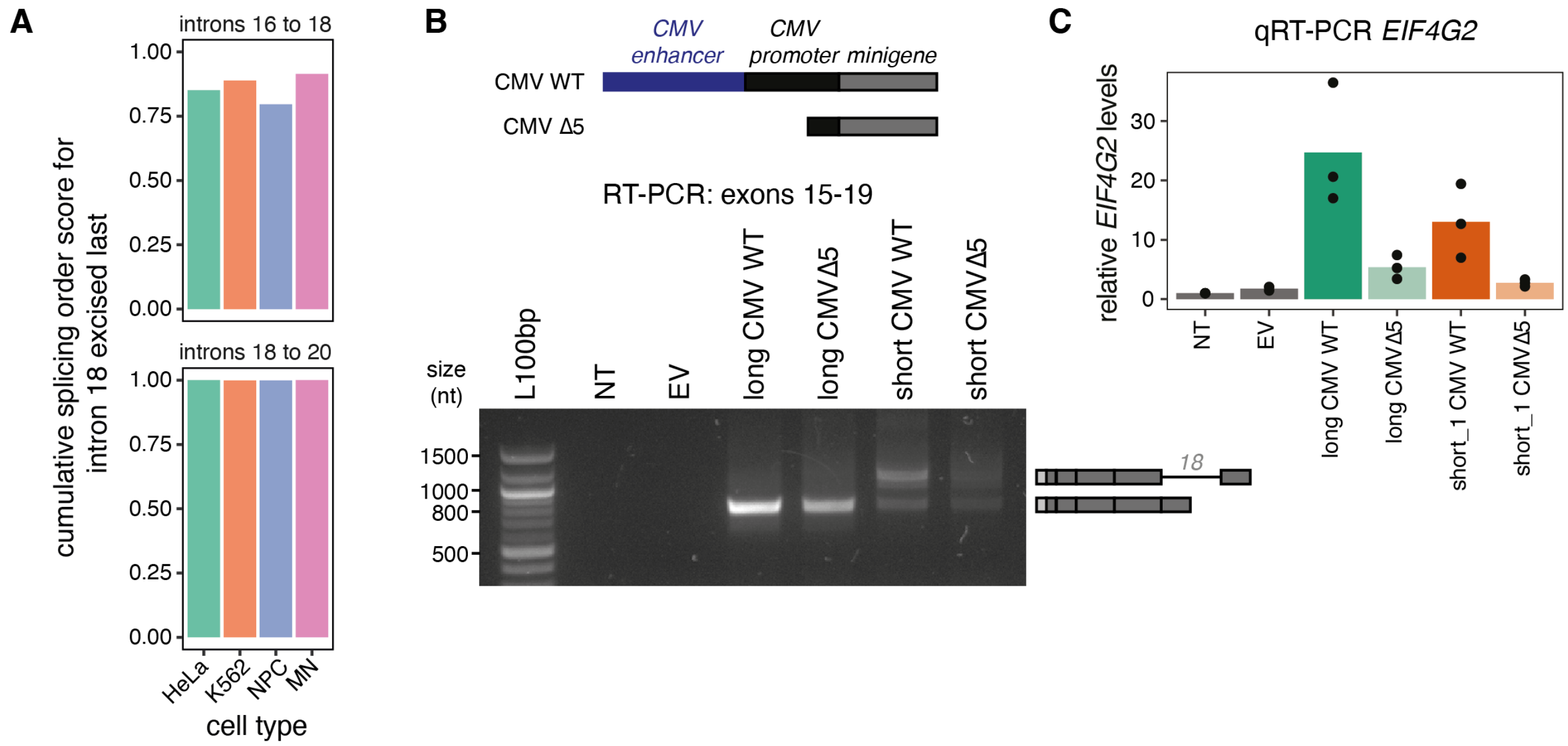

Figure S2. A) Cumulative splicing order score for all splicing orders in which intron 18 is excised last in the four cell types shown on the y-axis. Splicing orders were computed separately for introns 16 to 18 (top) and 18 to 20 (bottom). NPC: neuronal progenitor cells, MN: motor neurons. B) Top: Schematic representation of the Δ5 deletion of the CMV enhancer and part of the promoter (37). Bottom: Agarose gel electrophoresis of RT-PCR products obtained from expression of the indicated minigenes in HEK293T cells, using primers in the minigene-specific 5′UTR and exon 19, as in Fig. 2B. Products are schematized on the right. C) qRT-PCR of *EIF4G2* mRNA levels after expression of long and short_1 minigenes with or without the CMV Δ5 deletion. *EIF4G2* levels were normalized to *GAPDH* using the ΔΔCt method. Each dot represents one biological replicate. WT: wild-type, L100bp: 100 bp ladder, NT: no transfection, EV: empty vector.

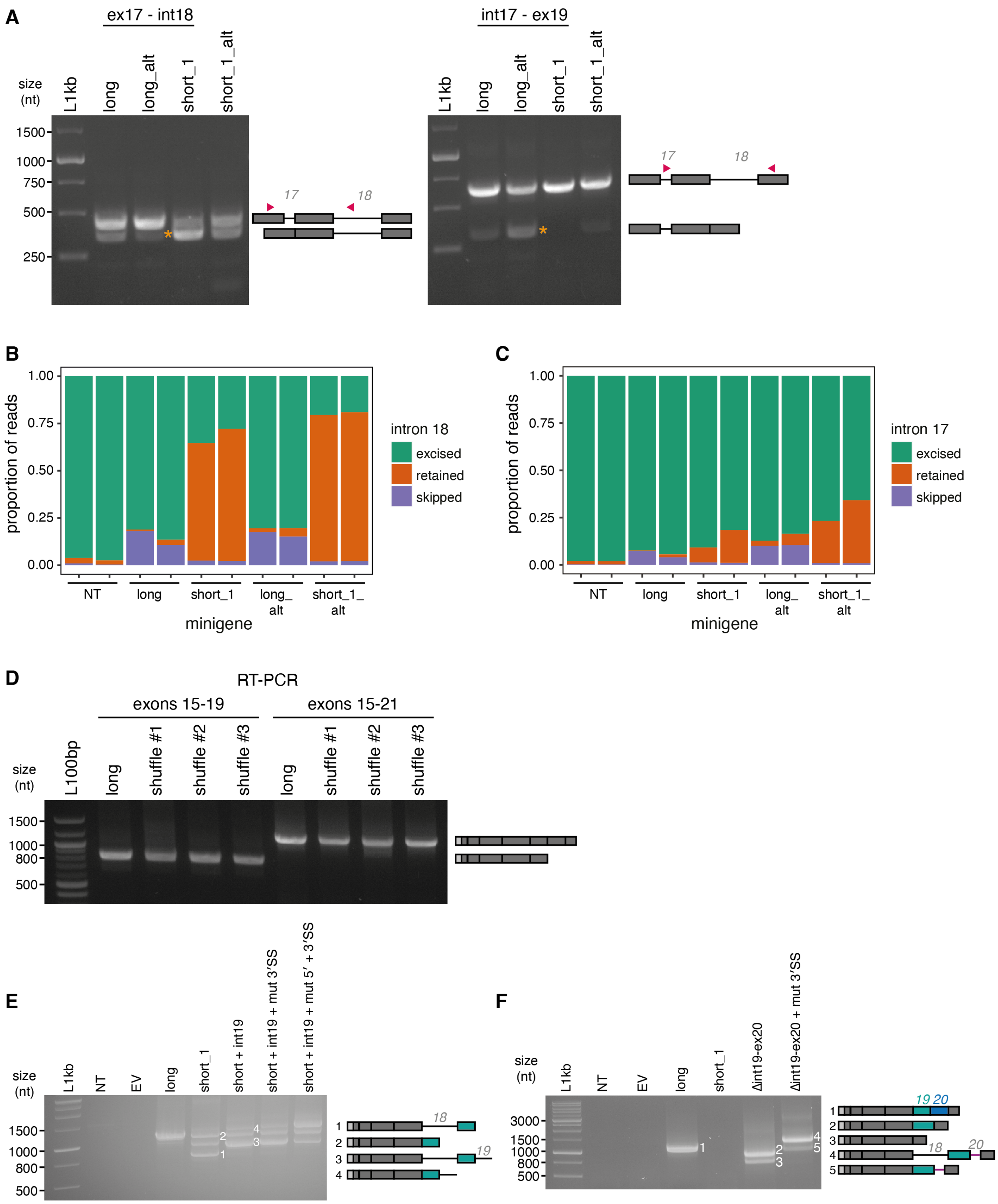

Figure S3. A) Agarose gel electrophoresis of RT-PCR products for splicing order analysis of *EIF4G2* introns 17 and 18 with the two primer pairs shown as dark pink arrowheads. Long_alt minigenes show increased levels of intron 17 being removed after intron 18 compared to long minigenes, as indicated by

the fainter band in the left panel and the darker band in the right panel (indicated by yellow asterisks). A similar but less pronounced pattern is observed for the short and short_alt minigenes. B) Nanopore sequencing of RT-PCR amplicons obtained from minigene transcripts, showing the proportion of reads in which intron 18 is excised, retained or its 3′ splice site is skipped. For the long, short_1, long_alt and short_1_alt minigenes, a forward minigene-specific primer was used with a reverse primer in exon 19 (dark pink arrowhead in A) or exon 21 (light pink arrowhead in A), respectively. RT-PCR in non-transfected cells (NT) was performed with primers in exons 15 and 21. Only reads spanning the full expected amplicons were included. Two biological replicates are shown for each condition. C) Same as B), but for intron 17. D) Agarose gel electrophoresis of RT-PCR products obtained from expression of three minigenes with distinct randomly shuffled internal intron 19 sequences. PCR was performed with a forward minigene-specific primer and a primer in exon 19 (left four lanes, as in Fig. 2B) or in exon 21 (right four lanes, as in Fig. 1C). E) Agarose gel electrophoresis of RT-PCR products obtained from the expression of the indicated minigenes, schematized in Fig. 8A, using primers in the minigene-specific 5′ and 3′ regions. Products with and without intron 18 retention are numbered and schematized on the right. F) Agarose gel electrophoresis of RT-PCR products obtained from the expression of the indicated minigenes, using a forward minigene-specific primer and a primer in exon 21. The main products are numbered and schematized on the right. L100bp: 100 bp ladder, L1kb: 1 kb ladder, NT: no transfection, EV: empty vector; int: intron; ex: exon.
